# Characterization of adverse events to hydroxychloroquine, ivermectin, azithromycin and tocilizumab in patients hospitalized due to COVID-19 in a Peruvian Social Health Insurance hospital

**DOI:** 10.17843/rpmesp.2023.401.11563

**Published:** 2023-03-28

**Authors:** L. Yesenia Rodríguez-Tanta, Enrique Cachay Rojas, Fabián Fiestas Saldarriaga, Guisela Alva Lozada, Paola Fernández-Rojas, Raquel Delgado-Escalante

**Affiliations:** 1 Instituto de Evaluación de Tecnologías en Salud e Investigación - IETSI, EsSalud, Lima, Peru. Instituto de Evaluación de Tecnologías en Salud e Investigación - IETSI EsSalud Lima Peru; 2 Hospital Nacional Edgardo Rebagliati Martins, EsSalud, Lima, Peru. Hospital Nacional Edgardo Rebagliati Martins EsSalud Lima Peru; 3 Instituto Nacional de Salud, Lima, Peru. Instituto Nacional de Salud Lima Peru

**Keywords:** Pharmacovigilance, COVID-19, Hydroxychloroquine, Azithromycin, Ivermectin, Tocilizumab

## Abstract

**Objective.:**

To characterize the adverse events (AEs) related to the off-label use of hydroxychloroquine (HQ), azithromycin (AZI), tocilizumab (TOB) and ivermectin (IVM) for the treatment of COVID-19 in hospitalized patients.

**Materials and Methods.:**

We conducted a secondary cross-sectional analysis of the Peruvian Social Health Insurance (EsSalud) pharmacovigilance system database of AE notifications to HQ, AZI, TOB and IVM in the Edgardo Rebagliati Martins National Hospital from April to October 2020. Information was collected from digital medical records. We estimated AE reporting rates and evaluated their characteristics by drug type, time of occurrence, type by the affected organ-system, severity and causality.

**Results.:**

We identified 154 notifications describing a total of 183 AE possibly related to HQ, AZI, TOB and IVM; the reporting rate was 8%. The median time of AE occurrence was 3 days (IQR: 2-5). Most were cardiovascular events; prolongation of the QT interval was the most frequent. Hepatobiliary AEs were mainly associated with TOB. Most cases were moderate, however, 10.4% were severe.

**Conclusions.:**

We found AEs potentially associated with the use of HQ, AZI, TOB and IVM against COVID-19; cardiovascular events were the most frequent. Although AZI, HQ and IVM have known safety profiles, their use against COVID-19 could increase the occurrence of AE due to the risk factors inherent to this infection. Surveillance systems must be improved, especially those for TOB.

## INTRODUCTION

In Peru, as in other countries during the most critical periods of the SARS-CoV-2 pandemic, pharmaceutical products were widely used without sufficient scientific evidence on their benefits and harms, including hydroxychloroquine (HCQ), chloroquine, azithromycin (AZI), ivermectin (IVM), tocilizumab (TOB), among others ^1^^-^^3^. In fact, some media and public figures promoted the inappropriate off-label use of these drugs, which led to self-medication and overmedication; and with it, increased risk of serious adverse drug reactions (ADRs) ^4^.

Although the safety profile of these drugs is known for their approved clinical indications, off-label use for the prevention and/or treatment of SARS-CoV-2 infection could alter their safety profile. Off-label use means that a medication is being used to treat a condition for which it was not approved. This could be related to the clinical picture of infected patients (electrolyte imbalance, proinflammatory state, disseminated intravascular coagulation status), high and prolonged doses of these drugs, polypharmacy and drug interactions, which would increase the risk of adverse events, which in some cases could be avoidable ^5^.

The implementation of pharmacovigilance in the context of the SARS-CoV-2 pandemic was essential, due to the high inpatient and outpatient demand for pharmaceutical products with limited scientific evidence on their benefits and safety in the treatment of COVID-19 ^6^. According to the World Health Organization (WHO), pharmacovigilance is the science and activities related to the detection, evaluation, understanding and prevention of adverse drug events, also known as adverse reactions, or any other drug-related problems ^7^.

Worldwide, several pharmacovigilance studies during the SARS-CoV-2 pandemic reported adverse events, such as prolongation of the QT interval and arrhythmias due to the use of HCQ^8^^,^^9^, in addition to the synergic arrhythmogenic effects of the AZI/HQ combination ^10^^-^^14^. Likewise, neurological ^15^ and gastrointestinal ^16^ adverse events have been reported for ivermectin, and serious hepatic effects ^17^ and neutropenia, superinfections, reactivation of latent infections, hepatitis and cardiac abnormalities for TOB ^18^.

In Peru, the Ministry of Health (MINSA), through the technical document “Prevention, Diagnosis and Treatment of persons affected by COVID-19”, recommended that the prescription of some of the aforementioned drugs in mild, moderate and severe cases of COVID-19, emphasizing that possible adverse reactions to these drugs should be monitored, since the safety information available on this disease is limited ^19^. However, little has been done in this regard, therefore, information on adverse events to these products is scarce in Peru.

Thus, the safety profile of “off-label” medications for COVID-19 in the Peruvian population needs to be described. Therefore, this study aims to characterize all reports of suspected adverse reactions associated with HCQ, AZI, TOB and IVM prescribed for the treatment of patients hospitalized for COVID-19 at the Edgardo Rebagliati Martins National Hospital (HNERM) and admitted to the Institutional Reference Center for Pharmacovigilance and Technovigilance of EsSalud (CRI-EsSalud), from April to October 2020.

**KEY MESSAGES**

Motivation for the study. To determine the safety profile of drugs used to treat COVID-19 without scientific evidence in Peru, through pharmacovigilance.

Main findings. Most adverse events were cardiovascular and hepatobiliary conditions. Adverse events were found to be underreported by health professionals.

Implications. This study allows the characterization of adverse events related to “off-label” drugs used during the most critical periods of the SARS-CoV-2 pandemic and helps to understand the importance of the implementation of pharmacovigilance.

## MATERIALS AND METHODS

### Study design

We carried out a secondary cross-sectional analysis of the pharmacovigilance database of the Peruvian Social Health Insurance (EsSalud) of all adverse event (AE) notifications to HQ, AZI, TOB and IVM prescribed during hospitalization as “off-label” in the treatment of hospitalized patients by COVID-19 during the period April to October 2020. Only notifications from HNERM made by healthcare personnel were included. Notifications that did not meet the time sequence criterion, i.e., that the AEs occurred before the date of exposure to the drug and reports coming from other healthcare centers were not included

EsSalud, through the Institutional Referral Center for Pharmacovigilance and Technovigilance (CRI-EsSalud), is a member of the National Pharmacovigilance and Technovigilance System led by the General Directorate of Medications, Inputs and Drugs (DIGEMID). The CRI-EsSalud receives all notifications of AEs related to pharmaceutical products (known as suspected adverse drug reactions) made by health professionals from EsSalud healthcare centers nationwide, in accordance with institutional and national Pharmacovigilance and Technovigilance regulations.

### Procedures

*Validation of clinical information and classification of the AE reports*

The clinical information of each AE notification to HCQ, AZI, TOB and IVM registered in the pharmacovigilance database of the CRI-EsSalud during the study period was validated, completed and analyzed through the review of the digital medical records of the EsSalud Intelligent Health Service (ESSI).

We also extracted data regarding other variables that are not considered in the AE notification format: COVID-19 severity, oxygen saturation at emergency admission, mechanical ventilation, COVID-19 outcome, AE duration, clinical data on the AE (laboratory values, electrocardiogram results, among others) and AE outcome.

AE severity was assessed according to the criteria established by the Peruvian Pharmacovigilance and Technovigilance System (mild, moderate and severe) ^20^. The naming of each AE was standardized according to the lowest level of terminology (LLT) of the Medical Dictionary for Regulatory Activities (MedDRA) according to the system-organ classification (SOC).

*Estimation of AE reporting rate and related drug type*

In order to estimate the AE reporting rate, we obtained the total consumption of the aforementioned drugs and the total number of patients prescribed these products during the study period from the HNERM Pharmacovigilance Committee.

*Causality Assessment*

Causality was assessed to establish the degree of individual association between the AE and the drug suspected of causing it (HQ, AZI, TOB and IVM). We used the modified Karch and Lasagna causality algorithm, which assesses seven parameters: temporal sequence, prior knowledge, effect of drug withdrawal, re-exposure, existence of alternative causes, contributing factors and complementary explorations. The categories of this algorithm are: definite, probable, possible, conditional, improbable and unclassified ^20^: AEs that are classified as “confirmed” are categorized as adverse drug reactions (ADRs). It should be noted that signs, symptoms and complications of SARS-CoV-2 infection were considered as alternative causes according to their stages.

### Statistical analysis

Categorical variables were described using absolute frequencies and proportions, while the median and interquartile range (IQR) were used for continuous variables. In order to calculate the AE reporting rate, we considered the number of patients who reported at least one AE related to any of these drugs (HQ, AZI, TOB and IVM) out of the total number of patients who received any of these products during the study period and multiplied it by 100. All statistical analyses were conducted in the statistical program STATA version 15.0.

### Ethical criteria

This study was approved by the Specific Research Ethics Committee for COVID-19 of EsSalud. The code of the Health Research Projects Registry (PRISA) of the Peruvian National Institute of Health was 1439.

## RESULTS

Between April to October 2020, we identified 154 notifications of COVID-19 hospitalized patients who had at least one AE during “off-label” pharmacological treatment of COVID-19 prescribed during their hospital stay at HNERM. A total of 183 AEs were identified, because 18.8% of patients had more than one AE. The median age of the affected patients was 59 (IQR: 50-70) years and 70.8% were male (109 out of 154). We found that most of the affected patients had no history of ADR (Table 1).

Most cases were moderate (53%; 82 out of 154), however, 10.4% were severe. It is important to note that 38 people who had an AE died. It was not possible to determine to what extent the drugs contributed to death (Table 1).

**Table 1:** Characteristics of patients with at least one reported adverse event.

Characteristic		n (%)
Sex		
	Female	45 (29.2)
	Male	109 (70.8)
Age in years ^a^		59 (50-70)
COVID-19 severity		
	Mild	56 (36.6)
	Moderate	82 (53.0)
	Severe	16 (10.4)
SaO_2_ on admission to the emergency room ^b^		88 (16)
Mechanical ventilation		
	Yes	30 (19.5)
	No	124 (80.5)
History of ADR		
	Yes	10 (6.4)
	No	144 (93.6)
Medical history ^c^		
	Arterial hypertension	56 (36.4)
	Diabetes *mellitus*	30 (19.5)
	Obesity	46 (29.9)
	Another ^d^	113 (73.4)
	Not stated	21 (13.6)
	None	44 (28.6)
COVID-19 outcome		
	Recovered	116 (75.3)
	Died	38(24.7)
Treatment prior to hospitalization ^e^		
	Azithromycin	38 (24.7)
	Corticoids	27 (17.5)
	Antibiotics	21 (13.6)
	Ivermectin	12 (7.8)
	Hydroxychloroquine	4 (2.6)
	Anticoagulants	4 (2.6)
	Another	53 (34.4)
	None	56 (36.4)
Number of AEs		
	One AE	125 (81.2)
	More than one AE	29 (18.8)

AE: adverse event; SaO_2_: oxygen saturation; ADR: adverse drug reaction.

a Median (interquartile range) ^b^ mean (standard deviation), ^c^patients may have more than one condition, ^d^ includes cancer, HIV, chronic lung disease, depression, arterial insufficiency and renal insufficiency, ^e^ patients may have received more than one medication.

### AE reporting rate

A total of 1895 HNERM patients received at least one of the four drugs (AZI, HCQ, TOB, IVM) during the study period, with the AE reporting rate being 8%. Likewise, we obtained the AE reporting rate for each drug. TOB had the highest AE reporting rate with 15.5% (13 cases of TOB-related AE out of 84 patients who received TOB), followed by HCQ with 10.1% (65 out of 641), AZI with 4.9% (85 out of 1749) and IVM with 4.7% (12 out of 255). The median time to onset of AE was 3 days (IQR: 2-5).

### AE according to organ-system classification

**Figure 1:**
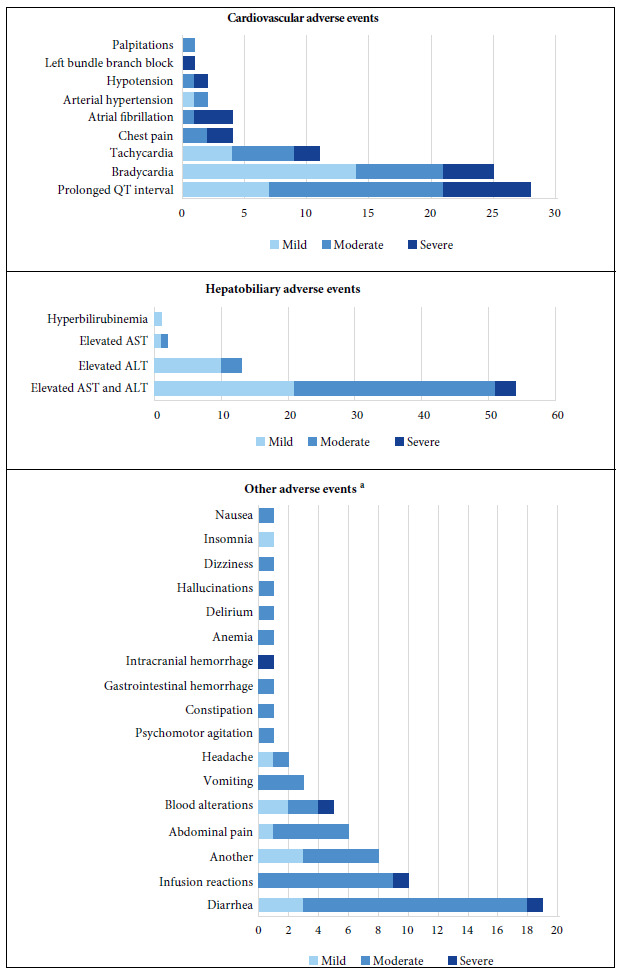
Adverse events by the lowest level terms according to system-organ classification and severity.

According to the SOC classification of AE, 39.5% (n=72) were cardiovascular, 35.5% (n=65) were hepatobiliary, 16.9% (n=26) corresponded to gastrointestinal events. The remaining 8.1% (n=17) belonged to other SOC classes (Figure 1). Table 2 details the characteristics by AE.

**Table 2:** Characteristics of the reported and analyzed adverse events.

Characteristics		Cardiovascular AE		Hepatobiliary AE		Gastrointestinal AE		Post-infusion AE		Hematological AE		Psychiatric Neurological AE	
		n=72	%	n=65	%	n=26	%	n=9	%	n=4	%	n=7	%
Sex													
	Female	19	26.6	19	28.6	10	38.7	-	-	1	25.0	2	28.6
	Male	53	73.4	46	71.4	16	61.3	9	100.0	3	75.0	5	71.4
Age (years)													
	≤50	26	36.7	19	28.6	2	6.5	4	44.4	1	25.0	-	-
	>50	46	63.3	46	71.4	24	93.5	5	55.6	3	75.0	7	100.0
Medical history ^a^													
	HT	27	38.0	12	18.6	6	22.6	1	11.1	2	50.0	4	57.1
	T2DM	12	16.5	8	12.9	3	9.7	3	33.3	1	25.0	2	28.6
	Respiratory (TBC)	2	2.5	7	11.4	-	-	2	22.2	1	25.0	-	-
	Another	20	27.9	12	18.6	6	22.6	1	11.1	2	50.0	2	28.6
	None	15	20.3	33	50.0	13	48.4	2	22.2	0	0	1	14.3
Severity													
	Mild	20	27.9	31	47.1	3	12.9	-	-	1	25.0	2	28.6
	Moderate	34	46.8	32	48.6	22	83.9	8	88.9	2	50.0	5	71.4
	Severe	18	25.3	2	4.3	1	3.2	1	11.1	1	25.0	-	-
Outcome													
	Recovered	49	68.0	56	85.7	25	96.7	8	88.9	3	75.0	5	71.4
	Died	23	32.0	9	14.3	1	3.3	1	11.1	1	25.0	2	28.6
Suspected drugs													
	AZI	17	24.1	13	20.0	19	77.4	-	-	1	25.0	5	71.4
	HCQ	12	16.5	10	15.7	1	3.2	-	-	-		-	
	AZI + HCQ	36	50.6	7	11.4	-	-	-	-	-		-	
	IVM (alone and combined)	-	-	9	14.3	2	6.5	-	-	-		2	28.6
	TOB	2	2.5	15	22.9	1	3.2	9	100.0	2	50.0	-	-
	Another ^b^	5	6.3	10	15.7	3	9.7	-	-	1	25.0	-	-
AE causality													
	Defined (ADR)	4	5.1	1	1.5	-	-	-	-	-	-	-	
	Probable	42	58.2	24	37.1	8	29.0	3	33.3	2	50.0	2	28.6
	Possible	19	26.6	26	40.0	15	58.1	6	66.7	1	25.0	5	71.4
	Conditional	7	10.1	14	21.4	3	12.9	-	-	1	25.0	-	-

AE: adverse event; HT: hypertension; T2DM: type 2 diabetes mellitus; TB: tuberculosis; AZI: azithromycin; HCQ: hydroxychloroquine; IVM: ivermectin; TOB: tocilizumab; ADR: adverse drug reaction.

a Patients may have more than one condition, ^b^ includes ranitidine, omeprazole, losartan, rendesivir, enoxaparin, ceftriaxone, linezolid, cefepime (possible interactions with AZT and HCQ).

Of the 72 reported cardiovascular AEs, 28 were cases of QT interval prolongation, of which 10 described QT interval ≥500 ms. Likewise, 37 cases of arrhythmias were reported (Figure 1). We found that 73.4% of the cases happened in men and 36.7% of the patients were 50 years of age or younger. In addition, 18 cases were severe and 23 patients died, but whether their death was causally related to AE could not be determined. We also found 36 reports of cardiovascular AEs describing the AZI/HCQ combination as a probable cause, 17 cases for AZI and 12 for HCQ. According to the causality assessment, 4 cases were confirmed and most were considered probable (58.2%).

Sixty-five hepatobiliary AEs (laboratory alterations) were identified, 64 cases had transaminitis (51 cases with elevated AST and ALT, 11 with elevated ALT and two with elevated AST) and one with hyperbilirubinemia. We found that 71.4% were men and 28.6% of the patients were 50 years old or younger. Two cases were severe and nine patients died. TOB and AZI were the most suspected to produce hepatobiliary AEs. In 45.7% of the cases, upon discontinuation of the suspected drugs, laboratory tests returned to baseline levels. Most cases were considered possible (40%) and probable (37.1%), however, one case was confirmed.

Gastrointestinal AEs were reported in 26 cases (15 cases of diarrhea and 11 of nausea, vomiting and abdominal pain). One case of diarrhea was severe because it extended the patient’s hospital stay. The notifiers indicated that AZI was possibly related to these AEs. Eighty percent of the cases with diarrhea subsided after discontinuation of AZI. Nine patients had AEs after intravenous administration of TOB. Of the total cases, one patient with severe COVID-19 died within six days of post-infusion reaction.

Four case reports showed hematological involvement (intracranial hemorrhage, anemia and hematological laboratory alterations). It is important to note that 3 cases showed potential drug interactions (TOB/linezolid, extended dose enoxaparin/fluoxetine, extended dose enoxaparin/methylprednisolone). One case was severe and fatal. Most cases were related to TOB and were considered probable.

Three cases of psychiatric symptoms (psychomotor agitation, delirium and hallucinations) and four neurological AEs were reported (two cases of headache, one of dizziness and one of insomnia). All occurred in patients older than 50 years of age. Most were moderate. AZI was suspected as the main cause of 71.4% of AEs. Reports of headache and insomnia describe MVI as the cause.

## DISCUSSION

This study characterized 154 reports of AEs associated with HCQ, AZI, TOB and IVM prescribed as “off-label” in the treatment of patients hospitalized for COVID-19 at HNERM. Most of the reports described male patients affected by at least one AE, this evidence is consistent with that described by other authors for the use of drugs without approved indication as treatment against COVID-19 ^8^^-^^10^^,^^21^^,^^22^. A possible explanation is that, worldwide, men had more severe COVID-19, and therefore, more risk of being hospitalized ^23^, of being exposed to pharmacological treatments against COVID-19 and consequently, they were more likely to have adverse reactions. It is important to mention that AEs to AZI were more frequent than to HCQ. However, most pharmacovigilance studies point to HCQ as the leading cause. This is possibly due to the fact that there was more AZI consumption than HCQ in EsSalud healthcare centers that reported at least one AE.

We identified 183 AEs from the 154 reports. Most of these were cardiovascular, with QT interval prolongation and arrhythmias standing out; the AZI/HCQ combination were the main cause of these AEs. One of the reasons for this statement is that the AZI/HCQ pharmacological interaction and its arrhythmogenic effects have been widely described during the COVID-19 pandemic ^8^^-^^14^^,^^24^. The arrhythmogenic mechanism of both drugs is explained by the blockade of cardiac voltage-dependent potassium channels, which would prolong cardiac repolarization, leading to ventricular arrhythmia ^25^. Additionally, AEs may occur due to drug-disease interaction, thus, the AZI/HCQ combination may prolong the QT interval, but this AE may be exacerbated by electrolyte imbalances in patients with COVID-19. Several drug regulatory agencies such as the European Medicines Agency (EMA) ^26^, the Spanish Agency for Medicines and Health Products (AEMPS) ^27^^,^^28^ and DIGEMID ^29^ warned of the risks of arrhythmias due to AZI/HCQ consumption, suggesting intensive cardiological monitoring if these drugs were prescribed.

Hepatobiliary (transaminitis) and gastrointestinal AEs were identified in this study. According to the causality assessment, most cases were possibly associated to TOB y AZI (score of 5-6 according to the causality algorithm by Karch and Lasagna), because COVID-19 was considered an alternative cause to transaminitis and diarrhea. Thus, previous studies describe that those two symptoms are the most frequent in patients with COVID-19 and hepatobiliary and gastrointestinal disorders, especially during stage 2 of the disease ^30^^,^^31^. However, it is important to note that the TOB medication guide describes both symptoms as frequent (≥1/100 to <1/10) ^32^ and that pharmacovigilance studies during the COVID-19 pandemic report hepatic AEs associated with TOB ^17^^,^^18^. For AZI, diarrhea is described as very frequent (≥1/10) and transaminitis as rare (≥1/1000 to <1/100) ^33^. Therefore, the association between this type of AEs and these pharmaceuticals, or with COVID-19 itself, cannot be ruled out.

The use of “off-label” medications can lead to errors during treatment which, in turn, could lead to the occurrence of AEs. In this study, we found AEs possibly associated with errors during treatment. Three cardiac AEs (QT interval prolongation, bradycardia and atrial fibrillation) were related to high doses and extended use of AZI (500 mg for seven days). We found one case of a subdural hematoma in a patient who received fluoxetine, enoxaparin (therapeutic dose for more than 21 days) and AZI 250 mg for 15 days. This drug interaction is considered dangerous and should be avoided ^34^. These cases had fatal outcomes. Additionally, we found a case of moderate thrombocytopenia in a patient receiving linezolid and enoxaparin. It should be noted that this AE is to be expected with both drugs. Initially, patients with COVID-19 were exposed to several drug combinations, which makes it difficult to identify the drug or drug association responsible for the AE. Therefore, the pharmacological characteristics of drugs should be considered before prescribing them. Pharmacovigilance should also be implemented.

The high hospital demand for pharmaceutical products with limited or no scientific evidence for the treatment of COVID-19 was an opportunity for expanding pharmacovigilance in Peru; however, it was not properly implemented ^35^. Both MINSA and the Instituto de Evaluación de Tecnologías en Salud e Investigación (IETSI) recommended pharmacovigilance for off-label treatment of COVID-19 ^36^. We found 154 AE reports at the HNERM, with a reporting rate of 8%. Although this is an unusual number of AE reports in seven months for the studied drugs, this may be due to substantial underreporting. Underreporting of suspected ADRs is a worldwide problem. The lack of awareness of the impact of reporting, the demanding workload of health professionals, especially at the beginning of the pandemic, are possible reasons for underreporting. Another important cause for underreporting is that health professionals consider pharmacovigilance as an administrative function rather than a healthcare activity. Pharmacovigilance contributes to patient safety; therefore, it must be considered as a clinical and healthcare activity by the professionals responsible for the direct care of patients with COVID-19.

The retrospective design was one of the main limitations of our study. Although we had access to electronic pharmacovigilance medical records, in some cases the information was incomplete, possibly due to the high patient demand. We did not access the physical medical records or nursing charts, the latter being important because it contains information regarding the doses of the drugs administered to patients. Likewise, although the modified Karch and Lasagna algorithm is supported by the Peruvian Pharmacovigilance System, the evaluation can be subjective when assessing parameters such as the existence of alternative causes, contributing factors and complementary evaluations. Furthermore, this algorithm only allows us to see the degree of association between the adverse event and the medication, but does not define causality, therefore we cannot assume that the drugs administered during hospitalization caused the adverse events, even more so when the information regarding the medication received outside the hospital was not available. Even with these limitations, this study did not only describe the HNERM reports from the EsSalud pharmacovigilance database, but also collected clinical information to make a causality assessment and describe the AE outcomes. In addition, this is one of the few studies that describes AE cases due to the use of “off-label” medication against COVID-19 in Latin America.

In conclusion, we found AEs associated with the widespread use of drugs against COVID-19 without scientific evidence during the beginning of the pandemic, with cardiovascular and hepatobiliary conditions standing out. AZI and the AZI/HCQ combination were the ones that were reported to be associated with most safety issues. However, AEs were also related to TOB and IVM. Most of the cases were moderate and the patients recovered. Additionally, AEs were found to be underreported, therefore, health professionals treating patients with COVID-19 should recognize the importance of pharmacovigilance of “off-label” drugs, mainly TOB, and thus contribute to patient safety.
