# Supplementary material for: Characterization of adverse events to hydroxychloroquine, ivermectin, azithromycin and tocilizumab in patients hospitalized due to COVID-19 in a Peruvian Social Health Insurance hospital
Source: Rev Peru Med Exp Salud Publica. 2023 Mar 28;40(1):16–24. doi: 10.17843/rpmesp.2023.401.11563 (PMC10953645; doi:10.17843/rpmesp.2023.401.11563)
Supplement: Translation

# Translation: Caracterización de eventos adversos a hidroxicloroquina, ivermectina, azitromicina y tocilizumab en pacientes hospitalizados por COVID-19 en un hospital del Seguro Social de Salud del Perú

**Authors:** L. Yesenia Rodríguez-Tanta, Enrique Cachay Rojas, Fabián Fiestas Saldarriaga, Guisela Alva Lozada, Paola Fernández-Rojas, Raquel Delgado-Escalante

**Affiliations:** 1 Instituto de Evaluación de Tecnologías en Salud e Investigación - IETSI, EsSalud, Lima, Perú.; 2 Hospital Nacional Edgardo Rebagliati Martins, EsSalud, Lima, Perú.; 3 Instituto Nacional de Salud, Lima, Perú.

**Keywords:** Farmacovigilancia, COVID-19, Hidroxicloroquina, Azitromicina, Ivermectina, Tocilizumab

## Abstract

**Objetivos.:**

Caracterizar los eventos adversos (EA) asociados a hidroxicloroquina (HQ), azitromicina (AZI), tocilizumab (TOB) e ivermectina (IVM) prescritos como «fuera de etiqueta» en el tratamiento de pacientes hospitalizados por la COVID-19.

**Materiales y métodos.:**

Se realizó un análisis secundario transversal de la base de datos del sistema de farmacovigilancia del Seguro Social de Salud del Perú (EsSalud) de las notificaciones de EA a HQ, AZI, TOB e IVM provenientes del Hospital Nacional Edgardo Rebagliati Martins de abril a octubre del 2020. Se revisaron las historias clínicas digitales, se estimaron las tasas de reporte de EA y se evaluaron sus características por tipo de fármaco, tiempo de aparición, tipo por órgano-sistema afectado, gravedad y causalidad.

**Resultados.:**

Se identificaron 154 notificaciones que describen un total de 183 EA posiblemente relacionados con HQ, AZI, TOB e IVM, siendo 8% la tasa de reporte de EA. La mediana de tiempo de aparición de EA fue de 3 días (RIC: 2-5). La mayoría fueron cardiovasculares, destacándose la prolongación del intervalo QT. Se observaron EA hepatobiliares principalmente asociados a TOB. La mayoría de los casos fueron moderados, no obstante, el 10,4% fue grave.

**Conclusiones.:**

Se identificaron EA potencialmente asociados al uso de HQ, AZI, TOB e IVM contra la COVID-19**,** siendo los más frecuentes los de tipo cardiovasculares. A pesar de que la AZI, HQ e IVM poseen perfiles conocidos de seguridad, su empleo en la COVID-19 podría incrementar la aparición de EA por los factores de riesgo propios de esta infección. Se sugiere reforzar la vigilancia, especialmente, de TOB.

## INTRODUCCIÓN

En el Perú, al igual que en otros países, durante los períodos más críticos de la pandemia por SARS-CoV-2 se usaron de manera extendida productos farmacéuticos sin suficiente evidencia científica sobre sus beneficios y daños, dentro de los que se destacan hidroxicloroquina (HCQ), cloroquina, azitromicina (AZI), ivermectina (IVM), tocilizumab (TOB), entre otros ^1^^-^^3^. De hecho, algunos medios de comunicación y figuras públicas promovieron el uso ambulatorio inapropiado de estos medicamentos, lo que conllevó a la automedicación y sobremedicación; y con ello, mayor riesgo de reacciones adversas a medicamentos (RAM) graves ^4^. 

Aunque el perfil de seguridad de estos medicamentos se conoce para sus indicaciones clínicas aprobadas; el uso «fuera de etiqueta» (*off-label*, en inglés) para la prevención y/o tratamiento de la infección por SARS-CoV-9 podría alterarlo. Se entiende por uso «fuera de etiqueta», a la utilización de un medicamento fuera de las indicaciones para el que fue aprobado. Algunas causas podrían estar relacionadas con el cuadro clínico de los pacientes infectados (desequilibrio electrolítico, estado proinflamatorio, el estado de coagulación intravascular diseminada), las altas y prolongadas dosis de estos fármacos, la polifarmacia y las interacciones farmacológicas, que aumentarían el riesgo de eventos adversos, que en algunos casos podrían ser evitables ^5^.

La implementación de la farmacovigilancia en el contexto de la pandemia producida por el SARS-CoV-2 es esencial, debido a la alta demanda hospitalaria y ambulatoria de productos farmacéuticos con limitada evidencia científica sobre sus beneficios y seguridad en el tratamiento de la COVID-19 ^6^. Según la Organización Mundial de la Salud (OMS), la farmacovigilancia es la ciencia y las actividades relacionadas a la detección, evaluación, comprensión y prevención de los eventos adversos de los medicamentos, también conocidos como reacciones adversas, o cualquier otro problema relacionado con ellos ^7^.

Diversos estudios de farmacovigilancia en el mundo, durante la pandemia por el SARS-CoV-2, mostraron eventos adversos, como la prolongación del intervalo QT y arritmias por el uso de HCQ ^8^^,^^9^, además de los efectos sinérgicos arritmogénicos de la combinación AZI/HQ ^10^^-^^14^. Asimismo, para el caso de ivermectina se han identificado eventos adversos neurológicos ^15^ y gastrointestinales ^16^ y, para TOB efectos hepáticos serios ^17^ y neutropenia, superinfecciones, reactivación de infecciones latentes, hepatitis y anormalidades cardiacas ^18^.

En el Perú, el Ministerio de Salud (MINSA), a través del documento técnico «Prevención, Diagnóstico y Tratamiento de personas afectadas por COVID-19» puso en consideración de médicos la prescripción de algunos de los citados medicamentos en casos leves, moderados y graves de COVID-19, resaltando que se debe monitorear las posibles reacciones adversas a estos fármacos, debido a que la información de seguridad disponible sobre esta enfermedad es limitada ^19^. Sin embargo, poco se ha realizado al respecto, por ende, la información sobre los eventos adversos a estos productos es escasa en el Perú.

En ese sentido, surge la necesidad de conocer el perfil de seguridad de los productos «fuera de etiqueta» para el tratamiento de la COVID-19 en población peruana. Por lo tanto, el presente estudio tiene por objetivo caracterizar todos los reportes de sospechas de reacciones adversas asociadas a HCQ, AZI, TOB e IVM prescritos en el tratamiento de pacientes hospitalizados por la COVID-19 en el Hospital Nacional Edgardo Rebagliati Martins (HNERM) y enviados al Centro de Referencia Institucional de Farmacovigilancia y Tecnovigilancia de EsSalud (CRI-EsSalud), durante el período de abril a octubre del 2020.

**MENSAJES CLAVE**

Motivación para realizar el estudio. Conocer el perfil de seguridad de medicamentos sin evidencia científica para el manejo clínico de la COVID-19, usados masivamente en el Perú, a través de la farmacovigilancia.

Principales hallazgos. Se identificaron eventos adversos de tipo cardiovascular y hepatobiliar, principalmente. Se observó un subreporte de eventos adversos por parte de los profesionales de la salud.

Implicancias. Este estudio permite caracterizar los eventos adversos de medicamentos «fuera de etiqueta» usados en los períodos más críticos de la pandemia por SARS-CoV-2 y reconocer la importancia de la implementación de la farmacovigilancia.

## MATERIALES Y MÉTODOS

### Diseño del estudio

Se realizó un análisis secundario transversal de la base de datos de farmacovigilancia del Seguro Social de Salud del Perú (EsSalud) de todas las notificaciones de eventos adversos (EA) a HQ, AZI, TOB e IVM prescritos durante la hospitalización como «fuera de etiqueta» en el tratamiento de pacientes hospitalizados por la COVID-19 durante el periodo de abril a octubre del 2020. Se incluyeron únicamente las notificaciones provenientes del HNERM que fueron hechas por el personal de la salud. Se excluyeron las notificaciones que no cumplían con el criterio de secuencia temporal, es decir, que los EA ocurrieran antes de la fecha de exposición al producto farmacéutico y aquellos reportes provenientes de otros centros asistenciales.

EsSalud, a través del Centro de Referencia Institucional de Farmacovigilancia y Tecnovigilancia (CRI-EsSalud), es integrante del Sistema Nacional de Farmacovigilancia y Tecnovigilancia liderado por la Dirección General de Medicamentos Insumos y Drogas (DIGEMID). El CRI-EsSalud recibe todas las notificaciones de EA relacionados a productos farmacéuticos (conocidos como sospechas de reacciones adversas a medicamentos) realizadas por los profesionales de la salud de los centros asistenciales de EsSalud a nivel nacional, de acuerdo con la normativa institucional y nacional de Farmacovigilancia y Tecnovigilancia.

### Procedimientos

*Validación de información clínica y clasificación de los reportes de EA*

Se validó, completó y analizó la información clínica de cada notificación de EA a HCQ, AZI, TOB e IVM registrada en la base de farmacovigilancia del CRI-EsSalud durante el periodo de estudio a través de la revisión de las historias clínicas digitales del Servicio de Salud Inteligente de EsSalud (ESSI).

Asimismo, se extrajo datos de otras variables que no son consideradas en el formato de notificación de EA: severidad de COVID-19, saturación de oxígeno a la admisión de la emergencia, ventilación mecánica, desenlace del COVID-19, duración del EA, datos clínicos sobre el EA (valores laboratoriales, resultados de electrocardiograma, entre otros) y desenlace del EA.

Se valoró la gravedad de los EA de acuerdo con los criterios establecidos por el Sistema Peruano de Farmacovigilancia y Tecnovigilancia (leve, moderada y grave) ^20^. Se estandarizó la denominación de los EA según el nivel más bajo de terminología (LLT, por sus siglas en inglés) del Diccionario Médico para Actividades Reguladoras (MedDRA) de acuerdo a la clasificación del sistema-órgano (SOC, por siglas en inglés).

*Estimación de la tasa de reporte de EA y tipo de fármaco relacionado*

Para estimar la tasa de reporte de EA se solicitó al Comité de Farmacovigilancia del HNERM el consumo total de los mencionados fármacos y el total de pacientes a los que se les prescribió estos productos, durante el periodo del estudio.

*Evaluación de la Causalidad*

Se evaluó la causalidad para establecer el grado de asociación individual entre el EA y el fármaco sospechoso de haberlo causado (HQ, AZI, TOB e IVM). Se utilizó el algoritmo de causalidad de Karch y Lasagna modificado, que valora siete parámetros: secuencia temporal, conocimiento previo, efecto del retiro del medicamento, reexposición, existencia de causas alternativas, factores contribuyentes y exploraciones complementarias. Las categorías de este algoritmo son: definida, probable, posible, condicional, improbable y no clasificada ^20^: Los EA que son clasificados como «definida» son catalogados como reacciones adversas a medicamentos (RAM). Se resalta que se ha considerado como causas alternativas los signos, síntomas y complicaciones de la infección por SARS-CoV-2 de acuerdo con sus estadios. 

### Análisis estadístico

Se realizó un análisis descriptivo de las variables categóricas usando frecuencias absolutas y proporciones, mientras que las variables continuas se muestran con la mediana y su rango intercuartílico (RIC). Para calcular la tasa de reporte de EA se consideró el número de pacientes para los que se notificó al menos un EA relacionado con cualquiera de estos medicamentos (HQ, AZI, TOB e IVM), sobre el total de pacientes a los que se les prescribió cualquiera de estos productos, durante el periodo de estudio y se multiplicó por 100. De la misma forma, se estimó la tasa de reporte de EA para HQ, AZI, TOB e IVM. Todos los análisis estadísticos fueron hechos en el programa estadístico STATA versión 15.0.

### Criterios éticos

Se tuvo la aprobación del Comité de Ética en Investigación Específico para la COVID-19 de EsSalud. El código del Registro de Proyectos de Investigación en Salud (PRISA) del Instituto Nacional de Salud de Perú fue 1439.

## RESULTADOS

Entre abril a octubre del 2020, se identificaron 154 notificaciones de pacientes hospitalizados por COVID-19 que presentaron al menos un EA durante el tratamiento farmacológico «fuera de etiqueta» de COVID-19, prescrito durante su estancia hospitalaria en el HNERM. Se identificaron un total de 183 EA, debido a que el 18,8% de los pacientes presentó más de un EA. La mediana de la edad de los pacientes afectados fue de 59 (RIC 50-70) años. El 70,8% fueron varones (109 de 154). Se observó que la mayoría de los afectados no tenían antecedentes de RAM (tabla 1).

**Tabla 1:** Características de los pacientes con al menos un evento adverso descrito en los reportes.

Característica		n (%)
Sexo		
	Femenino	45 (29,2)
	Masculino	109 (70,8)
Edad en años ^a^		59 (50-70)
Severidad de COVID-19		
	Leve	56 (36,6)
	Moderado	82 (53,0)
	Grave	16 (10,4)
SaO_2_ en la admisión de emergencia ^b^		88 (16)
Ventilación mecánica		
	Sí	30 (19,5)
	No	124 (80,5)
Antecedentes de RAM		
	Sí	10 (6,4)
	No	144 (93,6)
Antecedentes clínicos ^c^		
	Hipertensión arterial	56 (36,4)
	Diabetes *mellitus*	30 (19,5)
	Obesidad	46 (29,9)
	Otros ^d^	113 (73,4)
	No indica	21 (13,6)
	Ninguno	44 (28,6)
Desenlace de la COVID-19		
	Recuperado	116 (75,3)
	Falleció	38(24,7)
Tratamiento farmacológico previo a la hospitalización ^e^		
	Azitromicina	38 (24,7)
	Corticoides	27 (17,5)
	Antibióticos	21 (13,6)
	Ivermectina	12 (7,8)
	Hidroxicloroquina	4 (2,6)
	Anticoagulantes	4 (2,6)
	Otros	53 (34,4)
	Ninguno	56 (36,4)
Número de EA		
	Un EA	125 (81,2)
	Más de un EA	29 (18,8)

EA: evento adverso; SaO_2_: saturación de oxígeno; RAM: reacción adversa a medicamentos.

a Mediana (rango intercuartílico), ^b^ media (desviación estándar), ^c^ los pacientes pueden tener más de un antecedente clínico, ^d^ incluye al cáncer, VIH, enfermedad pulmonar crónica, depresión, insuficiencia arterial e insuficiencia renal, ^e^ los pacientes pueden tener más de un tratamiento.

La mayoría de los reportes fueron moderados (53%; 82 de 154), no obstante, se evidenció que el 10,4% fue grave. Es de notar que, 38 personas que tuvieron un EA fallecieron. No es posible determinar en este estudio en qué medida los medicamentos contribuyeron causalmente en dicha muerte (Tabla 1). 

### Tasa de reporte de EA

Un total de 1895 pacientes del HNERM recibieron al menos uno de los cuatro productos farmacéuticos (AZI, HCQ, TOB, IVM) durante el periodo del presente estudio, siendo la tasa de reporte de EA de 8%. Asimismo, se ha obtenido la tasa de reporte de EA por cada fármaco de interés prescrito, encontrando que TOB tuvo la mayor tasa de reporte de EA con 15,5% (13 casos de EA relacionado con TOB de 84 pacientes que recibieron TOB), seguido de HCQ con 10,1% (65 de 641), AZI con 4,9% (85 de 1749) e IVM con 4,7% (12 de 255). Se observó que la mediana de tiempo de aparición del EA fue 3 días (RIC 2-5).

### EA según la clasificación del sistema-órgano

Según la clasificación SOC de los EA, el 39,5% (n=72) fueron cardiovasculares, el 35,5% (n=65) fueron hepatobiliares, el 16,9% (n=26) corresponde a los gastrointestinales. El 8,1% (n=17) restante pertenece a otros SOC (Figura 1). En la Tabla 2 se detallan las características por EA.

**Tabla 2:** Características de los eventos adversos reportados y analizados.

Características		EA cardiovasculares		EA hepatobiliares		EA gastrointestinales		EA Posinfusionales		EA hematológicos		EA psiquiátricos neurológicos	
		n=72	%	n=65	%	n=26	%	n=9	%	n=4	%	n=7	%
Sexo													
	Femenino	19	26,6	19	28,6	10	38,7	-	-	1	25,0	2	28,6
	Masculino	53	73,4	46	71,4	16	61,3	9	100,0	3	75,0	5	71,4
Edad (años)													
	≤50	26	36,7	19	28,6	2	6,5	4	44,4	1	25,0	-	-
	>50	46	63,3	46	71,4	24	93,5	5	55,6	3	75,0	7	100,0
Antecedentes clínicos ^a^													
	HTA	27	38,0	12	18,6	6	22,6	1	11,1	2	50,0	4	57,1
	DM2	12	16,5	8	12,9	3	9,7	3	33,3	1	25,0	2	28,6
	Respiratorios (TBC)	2	2,5	7	11,4	-	-	2	22,2	1	25,0	-	-
	Otros	20	27,9	12	18,6	6	22,6	1	11,1	2	50,0	2	28,6
	Ninguno	15	20,3	33	50,0	13	48,4	2	22,2	0	0	1	14,3
Gravedad													
	Leve	20	27,9	31	47,1	3	12,9	-	-	1	25,0	2	28,6
	Moderado	34	46,8	32	48,6	22	83,9	8	88,9	2	50,0	5	71,4
	Grave	18	25,3	2	4,3	1	3,2	1	11,1	1	25,0	-	-
Desenlace													
	Recuperado	49	68,0	56	85,7	25	96,7	8	88,9	3	75,0	5	71,4
	Fatal	23	32,0	9	14,3	1	3,3	1	11,1	1	25,0	2	28,6
Medicamentos sospechosos													
	AZI	17	24,1	13	20,0	19	77,4	-	-	1	25,0	5	71,4
	HCQ	12	16,5	10	15,7	1	3,2	-	-	-		-	
	AZI + HCQ	36	50,6	7	11,4	-	-	-	-	-		-	
	IVM (sola y en combinación)	-	-	9	14,3	2	6,5	-	-	-		2	28,6
	TOB	2	2,5	15	22,9	1	3,2	9	100,0	2	50,0	-	-
	Otros ^b^	5	6,3	10	15,7	3	9,7	-	-	1	25,0	-	-
Causalidad del EA													
	Definido (RAM)	4	5,1	1	1,5	-	-	-	-	-	-	-	
	Probable	42	58,2	24	37,1	8	29,0	3	33,3	2	50,0	2	28,6
	Posible	19	26,6	26	40,0	15	58,1	6	66,7	1	25,0	5	71,4
	Condicional	7	10,1	14	21,4	3	12,9	-	-	1	25,0	-	-

EA: evento adverso, HTA: hipertensión arterial; DM2: diabetes *mellitus* tipo 2, TBC: tuberculosis; AZI: azitromicina; HCQ: hidroxicloroquina; IVM: ivermectina; TOB: tocilizumab; RAM: reacción adversa a medicamentos.

a Los pacientes pueden tener más de un antecedente clínico, ^b^ incluye a ranitidina, omeprazol, losartan, rendesivir, enoxaparina, ceftriaxona, linezolid, cefepime (posibles interacciones con AZT e HCQ).

De los 72 EA cardiovasculares, se reportaron 28 casos de prolongación del intervalo QT, de los cuales 10 describieron el intervalo QT ≥500 ms. Asimismo, se notificaron 37 casos de arritmias (Figura 1). El 73,4% fueron hombres y el 36,7% de los pacientes tuvieron 50 o menos años. Además, 18 casos fueron graves y 23 pacientes fallecieron, pero no se pudo determinar si su muerte fue causalmente relacionada con el EA. Se observó 36 reportes de EA cardiovasculares que describen a la combinación AZI/HCQ como sospechosa de haberlos producido, 17 a la AZI y 12 a la HCQ. De acuerdo con la evaluación de causalidad, cuatro casos resultaron definidos y la mayoría fue considerada probable (58,2%).

**Figura 1:** Eventos adversos por términos de nivel más bajo según la clasificación del sistema-órgano y severidad

Se identificaron 65 EA hepatobiliares (alteraciones laboratoriales), 64 casos se trataban de hipertransaminasemia (51 casos con TGO y TGP elevadas, 11 con TGP y dos con TGO elevadas) y uno de hiperbilirrubinemia. El 71,4% fueron hombres y el 28,6% de los pacientes tuvieron 50 o menos años. Se evidenciaron dos casos graves y nueve pacientes fallecieron. TOB y AZI fueron los más sospechosos de producir los EA hepatobiliares. En el 45,7% de los casos, al suspenderse el uso de los medicamentos sospechosos, los valores laboratoriales volvían a sus niveles basales. La mayoría de los casos fueron considerados posibles (40%) y probables (37,1%), no obstante, se observó un caso definido.

Se evidenciaron 26 reportes de EA gastrointestinales (15 casos de diarrea y 11 de náuseas, vómitos y dolor abdominal). Un caso de diarrea fue grave por prolongar la estancia hospitalaria del paciente. Los notificadores indicaron a AZI como el medicamento posiblemente relacionado con estos EA. El 80% de los casos de diarrea cedieron tras suspender AZI. Nueve pacientes presentaron EA luego de la administración intravenosa de TOB. Del total de casos, un paciente con la COVID-19 grave falleció después de seis días de presentar la reacción posinfusional.

Se recibieron cuatro reportes de casos con compromiso hematológico (hemorragia intracraneana, anemia y alteraciones laboratoriales hematológicas). Se resalta tres casos de potenciales interacciones farmacológicas (TOB/linezolid, enoxaparina dosis extendida/fluoxetina, enoxaparina dosis extendida/metilprednisolona). Un caso fue grave y fatal. La mayoría de los casos estuvo relacionada con TOB y fueron considerados probables.

Se notificaron tres casos de problemas psiquiátricos (agitación psicomotriz, delirio y alucinaciones) y cuatro EA neurológicos (dos casos de pacientes con cefalea, uno con mareo y un paciente con insomnio). Todos se presentaron en mayores de 50 años. La mayoría fueron valorados como moderados. Se sospechó de AZI como el principal causante del 71,4% de estos EA. Los reportes de cefalea e insomnio describen a la IVM como su causante. 

## DISCUSIÓN

El presente estudio caracteriza 154 reportes de EA asociados a HCQ, AZI, TOB e IVM prescritos como «fuera de etiqueta» en el tratamiento de pacientes hospitalizados por la COVID-19 en el HNERM. Se evidenció que la mayor parte de los reportes describen pacientes varones afectados por al menos un EA, esta evidencia es consistente con lo descrito por otros autores por el uso de medicamentos sin indicación aprobada para el tratamiento de la COVID-19 ^8^^-^^10^^,^^21^^,^^22^^).^ Una posible explicación es que a nivel mundial los varones tuvieron más COVID-19 severo, por ende, más riesgo de ser hospitalizados ^23^, de ser expuestos a tratamientos farmacológicos contra la COVID-19 y consecuentemente, tienen más probabilidad de presentar reacciones adversas. Es importante mencionar que, se encontró más eventos adversos (EA) relacionados con AZI que a HCQ. No obstante, la mayoría de los estudios publicados sobre farmacovigilancia señalan a HCQ como principal responsable. Esto posiblemente se deba a que hubo más consumo de AZI que HCQ en los centros asistenciales de EsSalud que reportaron al menos un EA.

A partir de los 154 reportes se identificaron 183 EA. La mayoría de estos fueron cardiovasculares, destacando la prolongación del intervalo QT y arritmias, siendo la combinación de AZI/HCQ la principal causa de estos EA. Una de las razones para esta afirmación es que la interacción farmacológica AZI/HCQ y sus efectos arritmogénicos han sido ampliamente descritos durante la pandemia de la COVID-19 ^8^^-^^14^^,^^24^^).^ El mecanismo arritmogénico de ambas drogas se explica por el bloqueo de los canales de potasio dependientes del voltaje cardiaco, lo que prolongaría la repolarización cardiaca, conllevando a arritmias ventriculares ^25^. Adicionalmente, se debe tener en cuenta que, los EA pueden presentarse debido a la interacción fármaco-enfermedad, así, la combinación AZI/HCQ puede prolongar el intervalo QT, pero este EA puede verse exacerbado por los desequilibrios electrolíticos de los pacientes con la COVID-19. Varias agencias reguladoras de medicamentos como la Agencia Europea de Medicamentos (EMA, por sus siglas en inglés) ^26^, la Agencia Española de Medicamentos y Productos Sanitarios (AEMPS) ^27^^,^^28^ y la DIGEMID ^29^ advirtieron los riesgos de arritmias por consumo de AZI/HCQ, sugiriendo un monitoreo intensivo cardiológico en caso de que se prescriban.

En el presente estudio, se identificaron EA hepatobiliares (aumento de transaminasas) y EA gastrointestinales. De acuerdo con la evaluación de causalidad de estos casos, la mayoría resultó «posiblemente asociado» principalmente a TOB y AZI (puntaje 5-6 según el algoritmo de causalidad de Karch y Lasagna). Esto debido a que la COVID-19 fue considerada una causa alternativa a la hipertransaminasemia y a la diarrea. Así, estudios describen a ambos síntomas como los más frecuentes en el grupo de trastornos hepatobiliares y gastrointestinales en pacientes con la COVID-19, especialmente, en la fase 2 de la enfermedad ^30^^,^^31^. No obstante, es importante reconocer que, la ficha técnica de TOB describe a ambos síntomas como frecuentes (≥1/100 a <1/10) ^32^ y que estudios de farmacovigilancia durante la pandemia de la COVID-19 reportan EA hepáticos asociados a TOB ^17^^,^^18^. Para el caso de AZI, la diarrea es descrita como muy frecuente (≥1/10) y la hipertransaminasemia como poco frecuente (≥1/1000 a <1/100) ^33^. Por lo tanto, no se descarta la asociación entre estos EA con esos productos farmacéuticos, ni con la misma COVID-19. 

El uso de medicamentos «fuera de etiqueta» puede dar lugar a errores de medicación que, a su vez, podrían conllevar a la aparición de EA. En el presente análisis se evidenciaron EA posiblemente asociados a errores de medicación. Tres EA cardiacos (prolongación del intervalo QT, bradicardia y fibrilación auricular) relacionados a altas y prolongadas dosis de AZI (500 mg por siete días). Un caso de un hematoma subdural en un paciente expuesto a fluoxetina, enoxaparina (dosis terapéutica por más de 21 días) y AZI 250 mg por 15 días. Esta interacción farmacológica es considerada seria y debe ser evitada ^34^. Estos casos tuvieron desenlaces fatales. Adicionalmente, se observó un caso de trombocitopenia moderada en una paciente que recibía linezolid y enoxaparina. Se debe tener en cuenta, que este EA es esperable en ambos medicamentos. Inicialmente, los pacientes con la COVID-19 estuvieron expuestos a combinaciones farmacológicas variadas, lo que dificulta la identificación del medicamento o asociación de medicamentos responsable de los EA. Por lo tanto, antes de prescribir los medicamentos se debe considerar las características farmacológicas de estos e implementar la farmacovigilancia.

La alta demanda hospitalaria de productos farmacéuticos con limitada o ninguna evidencia científica en el tratamiento contra la COVID-19 significó una oportunidad para el desarrollo de la farmacovigilancia en el Perú; sin embargo, su implementación estuvo limitada ^35^. Tanto el MINSA como el Instituto de Evaluación de Tecnologías en Salud e Investigación (IETSI) recomendaron realizar farmacovigilancia al tratamiento «fuera de etiqueta» contra la COVID-19 ^36^. En el presente estudio se evidenciaron 154 reportes de EA en el HNERM, calculándose una tasa de reporte del 8%. Aunque es un número inusual de reportes de EA realizados en siete meses para los medicamentos explorados, este número puede tener un subreporte sustancial. El subreporte de sospechas de RAM es un problema mundial. El desconocimiento del impacto de la notificación, la demandante carga laboral de los profesionales de la salud, especialmente al inicio de la pandemia, son razones de la baja notificación. Otra razón importante para el subreporte es que los profesionales de salud consideran a la farmacovigilancia como una actividad administrativa y no asistencial. Es fundamental que los profesionales responsables de la atención directa de los pacientes con la COVID-19 consideren a la farmacovigilancia como una actividad clínica y asistencial, que contribuya con la seguridad del paciente.

Como limitaciones del presente estudio se debe reconocer la naturaleza retrospectiva de los datos. Si bien se tuvo acceso a las historias clínicas electrónicas de farmacovigilancia, en algunos casos la información clínica fue incompleta, posiblemente, por la alta demanda de pacientes. No se tuvo acceso a las historias clínicas físicas ni a los kárdex de enfermería, este último documento es importante porque registra la información real de las dosis de medicamentos administrados a los pacientes. Asimismo, si bien, el algoritmo de Karch y Lasagna modificado es respaldado por el Sistema Peruano de Farmacovigilancia, la evaluación puede resultar subjetiva al valorar parámetros como la existencia de causas alternativas, factores contribuyentes y exploraciones complementarias. Además, este algoritmo solo permite ver el grado de asociación entre el evento adverso y el medicamento, pero no define causalidad, por lo que no podemos asumir que los medicamentos administrados durante la hospitalización causen los eventos adversos descritos, más aún cuando la información sobre la medicación ambulatoria de los pacientes no estaba descrita. Aún con estas limitaciones, este estudio no solo ha hecho una descripción de los reportes del HNERM registrados en la base de farmacovigilancia de EsSalud, sino que se ha recogido información clínica de los afectados para hacer una evaluación de causalidad y describir los desenlaces de los EA. Además, es uno de los pocos estudios que describe los casos de EA por el uso de tratamientos farmacológicos «fuera de etiqueta» contra la COVID-19 en Latinoamérica.

En conclusión, se identificó EA asociados al uso masivo de medicamentos contra la COVID-19 sin evidencia científica en los periodos iniciales de la pandemia, destacándose los problemas cardiovasculares y hepatobiliares. AZI y la combinación AZI/HCQ fueron los medicamentos con más problemas de seguridad notificados. No obstante, también se registraron EA relacionados con TOB e IVM. La mayoría de los casos fueron moderados y los pacientes se recuperaron. Adicionalmente, se pudo constatar un subreporte de EA, por lo tanto, es necesario que el profesional de la salud responsable de la atención directa de los pacientes con la COVID-19, reconozca la importancia de la farmacovigilancia de los medicamentos «fuera de etiqueta», principalmente para TOB, y de esta forma contribuir con la seguridad del paciente.
